# Supplementary figures and images for: An institutional analysis of graduate outcomes reveals a contemporary workforce footprint for biomedical master’s degrees
Source: PLoS One. 2020 Dec 7;15(12):e0243153. doi: 10.1371/journal.pone.0243153 (PMC7721154; doi:10.1371/journal.pone.0243153)

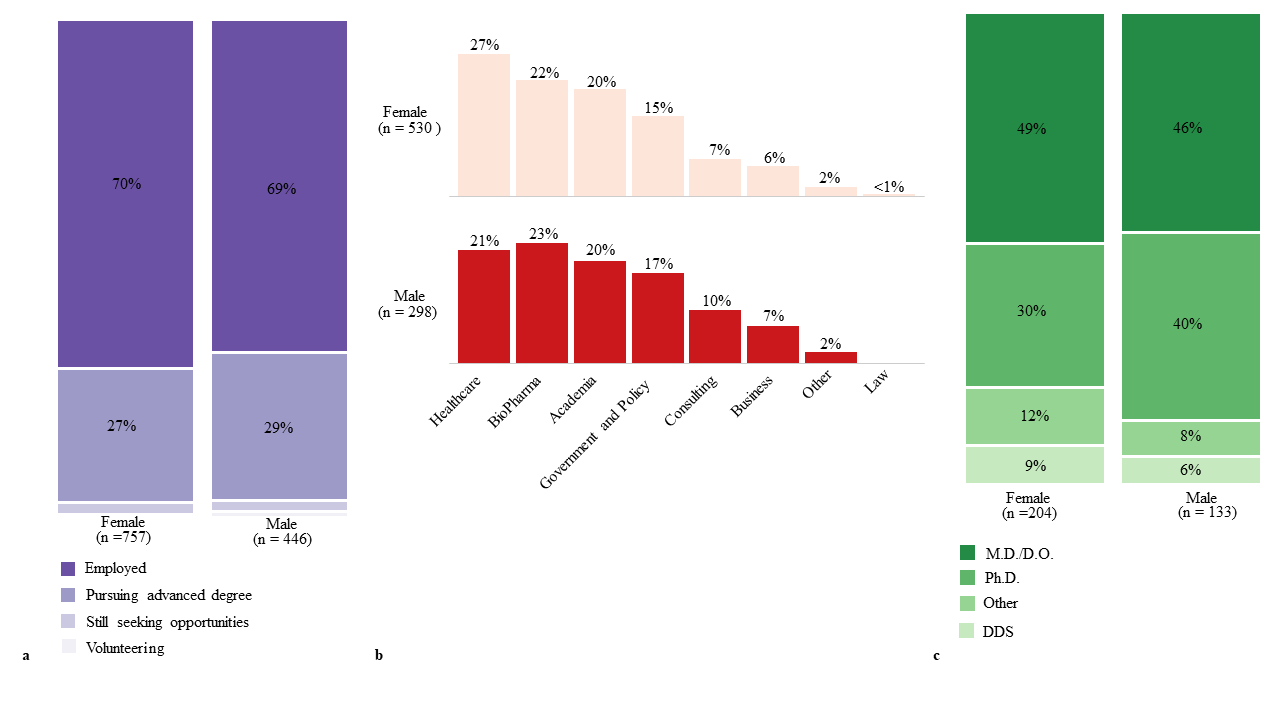

Supplement: S1 Fig — a) Outcome status by gender. b) Industry placement distribution of employed graduates by gender. b) Gender representation among graduates who pursued advanced degree programs after completing their M.S. degree. (TIF) [file pone.0243153.s002.tif]
